# Quality indicators of vocational schools: Partial least squares-SEM for small sample

**DOI:** 10.1016/j.mex.2025.103620

**Published:** 2025-09-10

**Authors:** Budi Susetyo, Anita Lie

**Affiliations:** a School of Data Science, Mathematics, and Informatics, IPB University, Indonesia; b Department of English Education, Faculty of Teacher Education, Universitas Katolik Widya Mandala Surabaya, Indonesia

**Keywords:** Structural equation model, Partial least squares, Indicators, School management, Teacher quality, Learning process, Learning outcome

## Abstract

This study aims to examine the relationship among the components of school management-governance, teacher quality, learning process, and learning outcome in vocational schools and evaluate the performance indicators in the accreditation instrument. Data were gathered from accreditation results of 32 vocational high schools with 44 measurement indicators from the National School Accreditation Board in Indonesia and analyzed through a Structural Equation Model using Partial Least Squares without normality assumptions. Results show that School Management-Governance significantly influences Teacher Quality (β = 0.714) and the Learning Process (β = 0.450), but not Learning Outcome. Teacher Quality affects the Learning Process (β = 0.479) but not Learning Outcome. The Learning Process has the strongest impact on Learning Outcome (β = 0.885), mediating other factors. A total of 8 measurement indicators are found insignificant, suggesting refinement of the accreditation instrument is needed. Insights drawn from this study will help other researchers and policy-makers, particularly those interested in educational quality measurement.

Some highlights of the proposed method are:

SEM-PLS is applied effectively to small-sample accreditation data without normality assumptions.

Learning Process is the strongest predictor of Learning Outcome, mediating other factors.

Only 36 of 44 accreditation indicators are significant, suggesting refinement is needed.

## Graphical abstract

**Figure:**
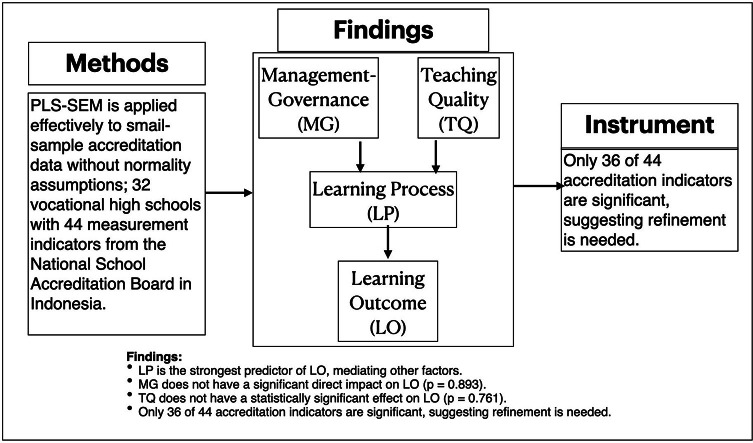

Specifications table

**Table:** 

Subject area	Mathematics and Statistics
More specific subject area	Applied Multivariate Analysis
Name of your method	structural equation model-partial least squares (PLS-SEM)
Name and reference of original method	J. F. Hair, G. T. M. Hult, C. M. Ringle, and M. Sarstedt, M, *A Primer on Partial Least Square Structural Equation Modeling (PLS-SEM).* United States of America (USA): SAGE, 2014.
Resource availability	Data from the accreditation results conducted by the National School Accreditation Board (BAN) of Indonesia

## Background

Several studies have shown that teaching quality is the central determinant of students' learning achievement, so teachers should be mandated to participate in competency development programs [[1], [2], [3]]. Jan [1] characterized the most effective learning procedure as one that provides a sufficient system for aiding teachers in enhancing their professional and teaching competencies. Excellent teachers are characterized as having strong teaching skills [4], communication skills [5], and a deep understanding of content knowledge [4], as well as demonstrating enthusiasm, care, and interpersonal skills [6]. Teachers should be capable of planning the lesson within the allotted time, setting expectations, and delivering effective learning. Based on the large data from the Indonesian National Assessment, Ping et al. [7] analyzed the relationship between perceptions of teaching quality and students’ achievement in literacy and numeracy and revealed the relevance of measuring teaching quality from the students’ and teachers’ perspectives to foster learning quality improvement.

As widely acknowledged, a good teacher performs great learning procedures and stages. Besides, the learning process should provide equal opportunities for every student to learn. During effective learning, students are taught to be aware of the importance of learning. Previous studies described learning as a voluntary activity, where each student always brings their own understanding, culture, values, social relations, assumptions, learning preferences, and learning motivations [8]. Furthermore, in their study of 193 teachers and 3457 students in 64 schools in Sumatera, Harjanto et al. [9] found a discrepancy between teachers’ and students’ perceptions of teaching quality. This discrepancy highlights the subjective nature of teaching quality and suggests that while professional development programs can enhance teachers’ self-efficacy, their actual effectiveness should also be assessed through student learning experiences and outcomes. In summary, a learning process includes knowledge, understanding, and skills in the social environment–through interaction with peers, teachers, and the wider group [10].

Furthermore, a school is a complex organization that requires capable principals with sufficient knowledge, competencies, and leadership. Thus, school principals should continuously upgrade their managerial and non-managerial skills as they carry substantial direct and indirect influences on students. Leithwood and Day [11] reported the seven most influential aspects of a school principal, two of which are directly related to the learning process and teaching effectiveness. In addition, Ko et al. [12] further described the importance of the leadership model in promoting orderly and excellent behavior, positive learning motivation, and culture in enhancing students' achievement. Harris [13] discusses the significance of contextual factors in leading school and system improvements. Hallinger [14] described that in accelerating the school quality, the principal should construct a collective vision, promote teachers' competency development, facilitate a conducive working culture, and involve the relevant stakeholders. These skills will multiply the positive effects on the teaching process.

Theoretically, Hoy and Smith [15] position the school principal as the most substantial factor in the school management and governance process. Accordingly, Smith [16] explained that the school principal should practice effective leadership styles. In this case, the school's innovation illustrates its ability to construct creative ideas for school improvement. There are three primary components of school innovation, namely a) the capacity to explore education strengthening, b) the ability to take opportunities, and c) the implementation of innovation to improve the school's performance and achievement. Thus, the entrepreneurial pattern should be adopted to provide effective learning. The school principal's entrepreneurial skills will aid them in facing the complexity and challenges of the school's environment, involving rapid changes, limited resources, and other factors, as well as the urgent obligation to prepare students for their future. These skills also enable the principal to construct substantial changes in the school [17]. These transformations and innovations are the influencing factors for a conducive environment. In addition, expectations for accountability should drive the school leadership to give space for school evaluators, even though the evaluation process may be tedious and time-consuming [18].

Ko et al. [12] asserted the clear effects of leadership on student's academic achievement through the teacher quality and teaching process, as well as promoting the proper climate and culture. The school's climate and culture are complex essential concepts for collaboration involving all stakeholders in the school. Therefore, the role of a school principal is influential in the school environment. The main task of the school principal is constructing and regulating the school culture [19]. A school with an excellent culture (high-performing school) carries characteristics. The school's culture and climate present significant impacts on the student's achievement [15], while the school's principal carries direct effects on the school's culture [11,20].

The education system should equip students with the necessary skills for solving more complex problems. Barron and Darling-Hammond [21] described that education should facilitate students' learning and regulate the demand for information, technology, work, and social transformation. Consequently, competent students are those with the skills to attain information, manage the information, and retell the information to the public. Lamb et al. [22] proposed a conceptual framework for students' successful learning containing five categories. First, the disposition consists of a sense of belonging, the ability to complete tasks using their competencies, a sense of efficacy, as well as a sense of hope and purpose. Second, intrapersonal skills include determination, self-control, and self-awareness. Third, interpersonal skills, including collaboration, communication, and leadership skills. Fourth, the engagement includes cognitive engagement, behavioral engagement, and emotional engagement. Fifth, cognitive skills, including reading, mathematics, and ICT skills, as well as creativity. These five categories are further specified into the nine essential skills: critical thinking, creativity, metacognition, troubleshooting, collaboration, motivation, self-efficacy, consciousness, and preservation.

In addition, education should equip students with global competencies. The recent global world has grown to be more multicultural, demanding everyone to see problems from different perspectives, as well as respect different opinions, viewpoints, and values. PISA defines global competence as the capacity to analyze global and cultural issues using critical procedures with various perspectives. This skill also correlates with the ability to get involved in an open interaction accurately and effectively. Further, PISA describes that this skill also covers the ability to understand essential local, global, and cultural issues, construct a dialog with the intercultural community, as well as attempting to realize collective welfare and sustainable development. Students with global competence are capable of using and incorporating various information to formulate questions, reasoning, analyzing, and examining global phenomena. After attaining the information, the students can analyze and evaluate the problems critically. Therefore, competent students acquire information and conduct reflective activities to voice out their opinion. Bialik et al. [23] group 21st-century skills into four domains, namely knowledge, skills, character, and metacognition. The knowledge group contains traditional knowledge, such as mathematics, language, and others, along with modern knowledge, such as robotics and entrepreneurship. The skills group includes communication, collaboration, creativity, and critical thinking skills. Meanwhile, the character group includes high curiosity, awareness (mindfulness), enthusiasm, resilience, ethics, and leadership. Metacognition puts forward the process of reflection across the three domains. Therefore, education must be able to prepare students to live in the 21st century with competencies, critical thinking, problem solving, communication, collaboration, and metacognition. Through education, students are required to develop their motivation, self-awareness, self-efficacy, and resilience. Thus, global competence is essential in the recent global era. This competence enables individuals to analyze global and intercultural issues using a critical thinking process. This competency also concerns the ability to engage in open, appropriate, and effective interactions with other people from different backgrounds based on mutual respect for human dignity. The required competencies to participate in the 21st century indicate the need for the school to reform its learning process. Consequently, the teacher, as the spearhead of successful learning in the classroom, must have the ability to translate student needs into the learning process. The school's principal also has to support teachers in carrying out their roles in the classroom.

Based on the theoretical framework above, it is pointed out that there are four interrelated components of the education process: School Principal's Management and Governance (MG), Teacher Quality (TQ), Learning Process (LP), and Learning Outcome (LO). This study aims to explore the relationship among the four components (school management and governance, teacher quality, learning process, and learning outcome) as quality indicators in vocational schools as revealed in the accreditation results using the structural equation model-partial least squares (PLS-SEM). In addition, this study aims to evaluate the performance of the 44 indicators in the accreditation instrument developed and used by the National School Accreditation Board (BAN-S/M) to accredit vocational high schools in Indonesia.

## Method details

This study uses data from accreditation results conducted by the National School Accreditation Board (BAN) in Indonesia. This study used the 2022 accreditation results data in 32 vocational high schools in East Java with 44 measurement indicators. Table 1 gives a summary of the Components in the Accreditation Instrument. Please see the Appendix for the 44 indicators.

**Table 1:** Latent variables, number of indicator variables, and their descriptions.

Components (latent variables)	Number of indicator variables	Descriptions of variable indicators
Learning Outcome (LO)	13	Measuring student character and competence and stakeholder satisfaction
Learning Process (LP)	9	Measuring the Quality of Learning in and outside the classroom; Classroom Learning Climate; and Utilization of Facilities and Infrastructure
Teacher Quality (TQ)	5	Measuring Teacher Competence; and Professional Development of Teacher Innovation and Creativity
Management and Governance (MG)	17	Measuring Vision and Mission Achievement; Principal Competence; School Culture; Community Involvement; Curriculum Management, Facilities and Infrastructure and Teachers and Education Personnel; Financing; Student affairs; and Quality Assurance

In 2020, the National School Accreditation Board (BAN-S/M) in Indonesia developed an instrument to accredit schools nationally. In the accreditation instrument, the learning outcome (LO) is measured with 13 indicators, the learning process (LP) with 9 indicators, the teacher quality (TQ) with 5 indicators, and the management and governance (MG) component with 17 indicators, so there are a total of 44 indicators. As the literature discussed above reveals, MG affects TQ, LP, and LO. TQ affects LP and LO, while LP also affects LO. Because MG, TQ, LP, and LO are latent variables that cannot be measured directly, their measurement is carried out through indicators in each latent variable. In the implementation of accreditation, the measurement of the 44 indicators is carried out by 2 assessors in each school during a two-day visit through interview methods, observations, and document checks. Then the assessor gives a score on a Likert scale from 1 (lowest) to 4 (highest).

The analysis method used in this study is a structural equation model with a parameter estimation approach using partial least squares. SEM analysis has two types of approaches, namely covariance-based structural equation modeling (CBEM) and variance-based structural equation modeling (VBSEM). CBSEM was first developed by Joreskog [24], Keesling [25], and Wiley [26], where the parameter estimation uses the maximum likelihood method. CBSEM requires the assumption of a double normal distribution and independence between residuals. Small samples can also provide poor parameter estimation results and statistical models and can even produce negative variance [27]. While VBSEM has two types, namely partial least squares path modeling (PLSPM) and generalized structured component analysis (GSCA). PLSPM was developed by Wold [28], while GSCA was developed by Hwang and Takane [29]. PLSPM and GSCA are quite powerful methods because they are not based on many assumptions. Referring to Hair et al. (2014), data does not have to be double normal distribution, the scale of the indicator variables can be in ordinal, interval, and ratio forms and the sample size does not have to be large [30]. Parameter estimation in PLS uses the fixed-point (FP) algorithm while GSCA uses alternating least squares (ALS). In this article, we will discuss parameter estimation with PLS (PLS-SEM) in more depth, then its application to accreditation data in cases where the sample size is not large and the indicator variables are ordinal.

Wold [28] introduced the Partial Least Squares (PLS-SEM) approach, which is a non-parametric statistical method, where in estimating the coefficients and testing the hypothesis does not require the assumption of a normal distribution of the latent variables [30]. Wold [28] further stated that PLS-SEM is a powerful method and is often referred to as soft modeling because it does not require the assumption of double normal distribution, freedom of error, and multicollinearity between exogenous variables. PLS can be used as a confirmation of theory (confirmatory) or (explanatory) to explain the existence or absence of relationships between latent variables that do not yet have a theoretical basis and suggest proportions for further testing [31]. PLS, according to Hair et al. [30], can also overcome serious problems if there is a singular matrix problem when estimating parameters. Un-identified, under-identified or over-identified problems will also never occur because PLS works on a recursive structural model.

**Fig. 1:**
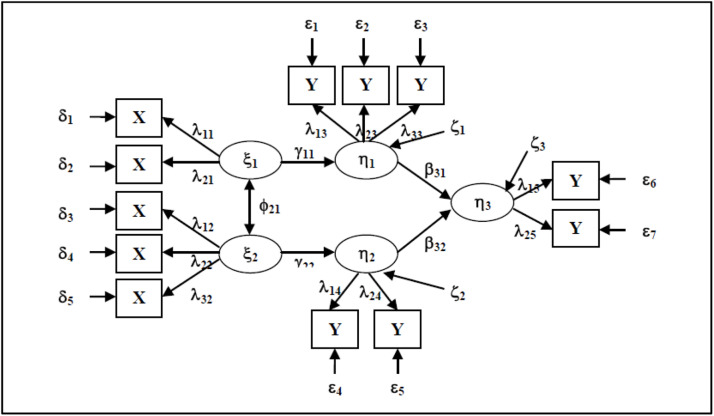
Diagram of the relationship between structural and measurement models.

Monecke and Leisch [32] and Ghozali and Latan [33] stated that PLS consists of three components, namely a structural model, a measurement model, and a weighting scheme. The structural model, or often called the inner model, is the relationship between latent variables, while the measurement model, or often called the outer model, shows how each indicator relates to its latent variables. The relationship among the four components is hypothesized as in Fig. 1. The relationship among latent variables is depicted in a structural model, while the relationship between latent variables and indicator variables is called a measurement model [34].

Notes:

() (ellipse) : latent variable (latent construct)

() (rectangle) : indicators (manifest variables)

ξ (ksi) : exogenous latent variable

η (eta) : laten endogenous latent variable

γ (gamma) : parameters to describe the direct relationship between exogenous variables and endogenous variables

β (beta) : parameters to describe the direct relationship of endogenous variables with other endogenous variables

ζ (zeta) : structural error that exists in an endogenous variable

ε (epsilon) : measurement error in indicators for endogenous latent variables

δ (delta) : measurement error in indicators for exogenous latent variables

λ (lambda) : loading factor, a parameter that describes the direct relationship between exogenous latent variables and their indicators

X : indicators related to exogenous latent variables

Y : indicators related to endogenous latent variables

Eqs. (1) and (2) are mathematical models of the relationship between latent variables and reflective indicators, while Eq. (3) is a model of the relationship between latent variables.

(1) $$y=\Lambda_{y}\eta +\varepsilon$$

(2) $$x=\Lambda_{x}\xi +\varepsilon$$

(3) η=βη+Γξ+ζ

The estimation of the parameters of Eqs. (1), (2), and (3) through the PLS algorithm is carried out through three stages, namely the Weight estimate stage, the Path estimation stage; and the Means calculation stage [31]. The Weight estimate stage aims to obtain the final estimate for each latent variable as a linear combination of the manifest variables by calculating the weights through an iteration process in Eq. (4):

(4) $$\xi_{j}=Y_{j}=\sum_{k}{w^{∼}}_{jk}X_{jk}$$

${w^{∼}}_{jk}$ is the outer weight scaled to provide the same $Y_{j}$ variance. The estimation approach is done through outside approximation for measurement models and inside approximation for structural models. Outside approximation aims to obtain a set of weights to estimate a latent variable that is able to calculate as much variance as possible from the indicators used and the constructs built. The inside approximation stage is carried out by considering the relationship between latent variables in the inner model to obtain a new approach from each latent variable that has been calculated in the outside approximation. The $Z_{j}$ internal estimation of the $\xi_{j}$ latent variable is formulated in Eq. (5)

(5) $$Z_{j}=\sum_{i;\beta_{ji}}e_{ji}Y_{ji}$$

$e_{ji}$ is the inner weight. The next stage is updating the outer weight. In the inside approximation stage, the information contained in the inner relations is entered into the latent variable estimation process. When the inside approximation stage is complete, the $Z_{j}$ internal estimates must be reviewed in relation to their indicators. This is done by updating the outer weight. In a reflexive relationship, each $w_{jk}$ weight is a regression coefficient of $Z_{j}$ on a simple regression between $X_{jk}$ and $Z_{j}$ (Eq. (6)).

(6) $$X_{jk}=w_{jk}Z_{j}$$

In the formative relationship, $Z_{j}$ is regressed on the indicator blocks related to the $\xi_{j}$ latent construct, and the $w_{jk}$weights as regression coefficients (Eq. (7)).

(7) $$Z_{j}=\sum_{k}w_{jk}X_{jk}$$

Therefore, $w_{j}$ is

(8) $$w_{j}={\left(X_{j}^{\prime }X_{j}\right)}^{-1}X_{j}^{\prime }Z_{j}$$

$X_{j}$ is a matrix containing the manifest variable $X_{jk}$ and $w_{j}$ is a weighting factor $w_{jk}$ (Eq. (8)).

The final part of the first stage is the convergence check. At each iteration stage *S* = 1,2,3,…, convergence is checked by comparing the outer weight value at the S-th iteration stage with the outer weight value at the (S-1)-th stage. Wold [28] suggested a limit $|{w^{∼}}_{jk}^{s-1}-{w^{∼}}_{jk}^{s}|<10^{-5}$ as a convergence limit. If it has converged, then the final estimated value of the latent variable is obtained (Eq. (9)).

(9) $$\xi_{j}=Y_{j}=\sum_{k}{w^{∼}}_{jk}^{new}X_{jk}$$

The second and third stages are the estimation of loading ${\lambda^{∼}}_{jk}$ and path coefficients ${\beta^{∼}}_{jk}$ for each inner model and outer model. The path coefficients in the structural model are estimated using the ordinary least square method in multiple regression $Y_{j}$ and $Y_{i}$ —the corresponding ones ((10), (11)).

(10) $$Y_{j}=\sum_{i}{\hat{\beta }}_{ji}Y_{i}$$

(11) $$\hat{\beta }={\left({Y^{\prime }}_{j}Y_{j}\right)}^{-1}{Y^{\prime }}_{j}Y_{j}$$

In the measurement model, the loading estimate depends on the type of relationship. In a reflexive relationship, the loading estimate is the regression coefficient from a simple linear regression $Y_{j}$ on $X_{jk}$ ((12), (13)).

(12) $$X_{ij}={\hat{\lambda }}_{jk}Y_{j}$$

(13) $${\hat{\Lambda }}_{j}={\left({Y^{\prime }}_{j}Y_{j}\right)}^{-1}{Y^{\prime }}_{j}X_{j}$$

The evaluation of the PLS-SEM model was conducted on both structural and the measurement models. The structural model was evaluated through the coefficient of determination (R-square) and the predictive relevance (Q-square) values. A better structural model is indicated by a higher R-square and Q-square value. Meanwhile, evaluation of the measurement model encompassed three critical aspects: convergent validity, discriminant validity, and composite reliability. The convergent validity pertains to the correlation between the indicator and its latent variable score. Ghozali and Latan (2012) stipulated that a factor loading value exceeding 0.6 satisfies the convergent validity requirement, while Chin (1998) suggested that a minimum threshold of 0.5 is adequate. Moreover, discriminant validity was assessed by juxtaposing the average variance extracted (AVE) for each latent variable against the correlations between them. According to Fornell and Lacker (1981), the condition for good discriminant validity is met if the square root of the AVE is greater than the correlation among the latent variables. Reliability testing within PLS-SEM is determined by the composite reliability value, with a range of 0.6 to 0.7 deemed satisfactory (Ghozali and Latan, 2012).

## Method validation

### Inter-component influence

**Fig. 2:**
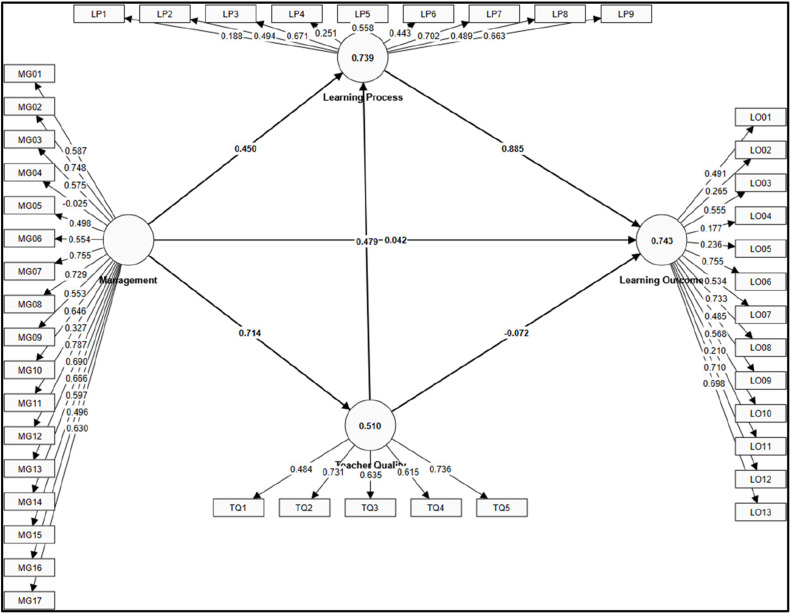
Results of parameter estimation using PLS-SEM.

This analysis was conducted using SmartPLS version 4.0.9.9, (SmartPLS GmbH, Bönningstedt, Germany) to estimate path coefficients and assess the measurement and structural models with bootstrapping procedure (5000 subsamples and significance level 5 %) and using path as weighting scheme. Fig. 2 presents the coefficients of the results of the inner model and outer model parameter estimation using the PLS approach. From the results of the data analysis, it can be concluded that the coefficient of determination (R Square) of the latent variable Teacher Quality is 51 %, meaning that the management carried out by school leaders is able to influence Teacher Quality by 51 %. Furthermore, School Management and Governance and Teacher Quality factors together are able to influence the quality of the Learning Process by 73.9 %. Meanwhile, the Management-Governance, Teacher Quality and Learning Process factors are able to influence Learning Outcome by 74.3 %. Apart from that, this model has a Q square of 0.97, so it can be concluded that this model also has a good level of predictive relevance.

Table 2 below illustrates the magnitude of the influence between components and the results of hypothesis testing. The School Management-Governance component has a significant direct influence and the largest is on Teacher Quality (0.714). The direct influence on the Learning Process is also significant with a coefficient of 0.450. However, this study shows that School Management-Governance does not have a direct effect on Learning Outcome. In addition, our results show that Teacher Quality also has a very significant influence on the Learning Process with a coefficient of 0.479, but Teacher Quality does not have a direct influence on Learning Outcome. From the results of this study, it can be seen that the factor that has the greatest influence on Learning Outcome is the Learning Process with a coefficient of 0.885. Management-Governance and Teacher Quality factors, although not directly influential, have an indirect influence on the Learning Process. These findings are in line with Leithwood, Jantzi, and Steinbach’s Mediated Effects Model [35] revealing that school leadership and teacher quality do not directly impact learning outcome but rather influence it through mediating factors like the learning process. This also aligns with the Constructivist Learning Theory [36] that learning outcome is primarily influenced by the learning process, which includes interaction with teachers, peers, and instructional materials. Setiawan et al. [37] in their research at the junior secondary school level and Hijrah et al. [38] at vocational high school also concluded that management standards have a direct influence on teacher standards, and process standards have a direct influence on graduation standards. In brief, Management-Governance and Teacher Quality improve the Learning Process, which in turn impacts Learning Outcome.

**Table 2:** Direct influence between latent variables.

Relation	Path Coefficient	T Statistics	P-Value	Decision
LP -> LO	0.885	3.200	0.001	Reject H0
MG -> LP	0.450	2.490	0.013	Reject H0
MG -> LO	0.042	0.135	0.893	Accept H0
MG -> TQ	0.714	7.523	0.000	Reject H0
TQ -> LP	0.479	2.958	0.003	Reject H0
TQ -> LO	−0.072	0.304	0.761	Accept H0

PLS-SEM analysis also produces an estimator of the coefficient of influence of the indicator variable on the latent variable and its test results. The results of this analysis can be useful to evaluate the accreditation instrument and whether the assessment items used to assess schools are adequate enough or not.

### Measurement indicators for school management-governance components

Table 3 is the coefficient of influence of indicator variables on management and governance components. Of the 17 indicator variables that describe the management and governance components in the accreditation instrument, there are 2 indicators that do not have a real influence, namely the indicator that measures whether the culture of interaction between schools and stakeholders has been running well (MG4) and the indicator that measures whether schools provide guidance and counseling services to support learning outcome and development of achievements (MG11). Both indicators from assessor measurements fail to distinguish variations between schools in describing the management and governance components.

**Table 3:** Outer Loading of Management and Governance.

Variable Indicators	Coefficient	T statistics	P values
MG1 <- MG	0.587	3.730	0.000
MG2 <- MG	0.748	8.390	0.000
MG3 <- MG	0.575	4.027	0.000
MG4 <- MG	−0.025	0.112	0.988
MG5 <- MG	0.498	2.461	0.002
MG6 <- MG	0.554	2.835	0.005
MG7 <- MG	0.755	10.159	0.000
MG8 <- MG	0.729	7.287	0.000
MG9 <- MG	0.553	3.303	0.001
MG10 <- MG	0.646	3.999	0.000
MG11 <- MG	0.327	1.734	0.083
MG12 <- MG	0.787	10.052	0.000
MG13 <- MG	0.690	6.959	0.000
MG14 <- MG	0.666	4.426	0.000
MG15 <- MG	0.597	3.592	0.000
MG16 <- MG	0.496	2.587	0.010
MG17 <- MG	0.630	4.339	0.000

A total of 15 indicators in the accreditation instrument can explain the school management and governance components clearly, namely indicators that measure whether schools develop and implement the school's vision and mission; the principal's competence in carrying out academic supervision; the principal's ability as a leader; schools are able to implement a safe, orderly, and comfortable culture; schools involve parents and the community; schools implement effective, efficient, and accountable management of teachers and education personnel; schools carry out good management of facilities and infrastructure; schools manage income and expenditure budgets transparently and accountably; schools organize student activity development to develop student interests and talents; schools implement Internal Quality Assurance; schools have networks/collaborations with the world of work and to improve the quality of learning; schools use their resources for practicum effectively and efficiently; and schools manage Special Job Fairs.

### Measurement indicators for teacher quality and learning process components

The Teacher Quality and Learning Process components are two components that are very closely related. The five indicators that measure the Teacher Quality components have a real influence. The five indicators measure whether teachers have the competence to prepare, implement and evaluate learning; teachers carry out continuous professional development; and teachers carry out internship activities in the world of work.

Of the 9 indicators that measure the Learning Process components, 7 indicators have a real influence, including those that measure whether the assessment of the Learning Process and Learning Outcome is used as a basis for improvement and is implemented systematically; remedial and/or enrichment programs are given to students who need them; teachers practice reading and writing literacy; teachers create a learning atmosphere that pays attention to safety, comfort, cleanliness, and makes it easy for students to learn; and the facilities and infrastructure available at schools/madrasas are optimally utilized in the learning process. Two insignificant indicators describe the components of the Learning Process, namely those that measure whether the process takes place actively by involving all students and developing high-level thinking skills; and whether students actively participate in learning and the learning atmosphere in the classroom is enjoyable. In contrast to the other seven indicators pointing to concrete performance such as teacher practices and learning atmosphere standards, the two indicators were found insignificant perhaps because they were phrased in such generic terms that assessors interpreted divergently.

### Measurement indicators for learning outcome components

Of the 13 indicators measured in the learning outcome component, 9 have a real influence and 4 are insignificant on the Learning Outcome component. The nine indicators include measuring whether students demonstrate disciplined behavior in various situations; students demonstrate resilient and responsible behavior in activities at school/madrasah; students demonstrate collaboration skills according to the characteristics of 21st-century skills; students demonstrate critical thinking and problem-solving skills according to the characteristics of 21st century; students demonstrate creativity and innovation skills according to the characteristics of 21st-century skills; students demonstrate the ability to express themselves and be creative in activities to develop interests and talents; students demonstrate increased learning achievement; graduates have competency certificates according to their expertise competencies; and graduates work/become entrepreneurs. The four indicators that do not significantly describe the Learning Outcome component include those that measure whether students demonstrate religious behavior in activities at school; students are free from bullying at school; students demonstrate communication skills according to the characteristics of 21st century skills; and stakeholders are satisfied with the quality of graduates. These four indicators were found to be insignificant probably because such statements referring to religious behaviors and stakeholders’ satisfaction were not deemed relevant to Learning Outcome.

Based on the outer loading presented in Tables 3, Table 4, Table 5, it can be concluded that the majority of indicators meet convergent validity because they have a value of 0.5 or more. Only 2 out of 17 indicators in the MG variable, 2 out of 9 indicators in the LP variable and 4 out of 13 indicators in the LO variable do not meet convergent validity, while all the indicators in the TQ variable are consistent with each other. Composite reliability for each of the four variables MG, LP, LO and TQ have values ​​of 0.907, 0.699, 0.830 and 0.668 respectively while the AVE values ​​for the four variables are 0.284, 0.274, 0.368 and 0.418 respectively. These show that the four latent variables have acceptable reliability.

**Table 4:** Outer Loading of Teacher Quality Components and Learning Process.

Variable Indicators	Coefficient	T statistics	P values
TQ1 <- TQ	0.484	2.341	0.019
TQ2 <- TQ	0.731	6.528	0.000
TQ3 <- TQ	0.635	3.246	0.001
TQ4 <- TQ	0.615	3.695	0.000
TQ5 <- TQ	0.736	4.691	0.000
LP1 <- LP	0.188	0.674	0.501
LP2 <- LP	0.494	2.450	0.014
LP3 <- LP	0.671	5.567	0.000
LP4 <- LP	0.251	0.909	0.363
LP5 <- LP	0.558	3.517	0.000
LP6 <- LP	0.443	0.223	0.020
LP7 <- LP	0.702	4.875	0.000
LP8 <- LP	0.489	2.072	0.038
LP9 <- LP	0.663	4.753	0.000

**Table 5:** Outer Loading of Learning Outcome.

Variable Indicators	Coefficient	T statistics	P values
LO1 <- LO	0.491	2.193	0.028
LO2 <- LO	0.265	1.112	0.234
LO3 <- LO	0.555	3.510	0.010
LO4 <- LO	0.177	0.637	0.524
LO5 <- LO	0.236	0.953	0.340
LO6 <- LO	0.755	9.340	0.000
LO7 <- LO	0.534	2.912	0.004
LO8 <- LO	0.733	8.390	0.000
LO9 <- LO	0.485	3.113	0.002
LO10 <- LO	0.568	2.753	0.006
LO11 <- LO	0.210	0.823	0.411
LO12 <- LO	0.710	5.186	0.000
LO13 <- LO	0.698	3.453	0.001

A total of eight accreditation indicators that are found insignificant in describing the three components of School Management-Governance (MG), Learning Process (LP), and Learning Outcome (LO) reveals that as a tool of education evaluation system, the accreditation system needs continuous improvement. As a matter of fact, at the time of writing this article the Indonesian National Accreditation Board has been revising its instruments to better assess schools and provide more useful feedback for quality improvement of the respective schools. This article will be sent to the Board and expected to provide valuable input on the accreditation performance indicators for schools at all levels.

## Discussion

The findings of this study provide significant insights into the quality indicators of vocational schools, particularly through the application of Structural Equation Modeling (SEM) analysis. The results demonstrate that the Learning Process (LP) has a strong and positive influence on Learning Outcome (LO), with a high path coefficient (0.885) and statistical significance (*p* = 0.001). This finding aligns with previous literature emphasizing the importance of an effective learning process in shaping student outcome [[1], [2], [3]]).

Additionally, Management and Governance (MG) positively affects LP (path coefficient = 0.450, *p* = 0.013), indicating that well-structured management contributes to the improvement of teaching and learning activities. However, MG does not have a significant direct impact on LO (*p* = 0.893), suggesting that its influence on Learning Outcome is mediated through LP rather than a direct effect. This result supports earlier research that underscores the indirect role of governance in education quality [11,14].

Furthermore, Teaching Quality (TQ) exhibits a strong positive relationship with LP (path coefficient = 0.479, *p* = 0.003) and MG (path coefficient = 0.714, *p* = 0.000). This highlights the essential role of effective teaching in fostering a conducive learning environment. However, TQ does not have a statistically significant effect on LO (*p* = 0.761), indicating that while high teaching quality contributes to LP, its impact on Learning Outcome may depend on additional mediating factors such as student engagement and curriculum relevance [4,12,18].

These results contribute to the ongoing discourse on vocational education quality by emphasizing the intricate relationships between Management and Governance, Teaching Quality, Learning Processes, and Learning Outcome. The study highlights that while some factors have direct effects, others play a more indirect but equally critical role. As Darling-Hammond and Adamson [39] indicate regarding the role of performance assessment in quality improvement, the integration of previous research reinforces the reliability of these findings and situates them within the broader context of vocational education improvement strategies.

### Implications

The findings of this study hold several key implications for policymakers, educators, and school administrators. First, since the Learning Process is the most significant factor influencing Learning Outcome, vocational schools should prioritize instructional quality by investing in professional development programs for teachers. Training should focus on innovative pedagogical strategies, competency-based learning, and student-centered approaches to enhance learning experiences.

Second, while Management-Governance does not directly impact Learning Outcome, its strong influence on Teaching Quality and the Learning Process suggests that effective school governance is crucial. School leaders should adopt strategic management practices, foster collaborative environments, and implement evidence-based policies that indirectly enhance student success.

Third, the indirect impact of Teacher Quality on Learning Outcome highlights the need for holistic teacher evaluations. Beyond assessing subject knowledge and instructional skills, teacher assessments should also consider their ability to create engaging and effective learning processes. Schools should also implement mentorship programs where experienced teachers guide new educators in improving their teaching methods.

All those three implications should be extended to non-vocational schools as well as primary and junior high schools. The method application of employing PLS-SEM in analyzing the relationship among the performance indicators in the accreditation instrument can and should also be conducted at other schooling levels to identify which components predict education quality and design a roadmap to plan and execute an education reform.

Finally, policymakers should consider revising accreditation and assessment frameworks to incorporate the complex interrelationships among School Management-Governance, Teacher Quality, Learning Processes, and Learning Outcome. Educational reforms should focus on strengthening school leadership capacities and creating policies that support a sustainable and high-quality learning environment in vocational schools. Furthermore, in line with the finding that 8 out of 44 indicators are not statistically significant, the Indonesian National School Accreditation Board should refine their assessment instruments and conduct a series of pilot administrations to ensure that all the indicators are valid and reliable.

## Conclusion

This study underscores the importance of key quality indicators in vocational education and their impact on Learning Outcome. The results confirm that a well-structured Learning Process significantly enhances student performance, reinforcing the necessity of improving teaching methodologies and curriculum design. Additionally, Management-Governance plays a crucial role in shaping learning environments, although its direct influence on Learning Outcome is limited. Teaching Quality, while important for Learning Processes, does not independently drive student success, suggesting that additional factors must be considered for a holistic improvement approach.

Given these findings, vocational institutions should focus on enhancing their management systems and pedagogical strategies to create an effective learning environment. Future research should explore additional mediating variables, such as student engagement and industry collaboration, to provide a more comprehensive understanding of vocational education quality indicators.

## Limitations

Further research on mediating variables like student engagement and industry collaboration is needed for a more comprehensive understanding of vocational education quality indicators.

## CRediT author statement

**Budi Susetyo**: Conceptualization, methodology, data curation, formal analysis, and writing—original draft preparation. **Anita Lie**: data and analysis validation, review and editing, project administration, and final manuscript approval.

Supplementary material and/or additional information [OPTIONAL]: None.

## Declaration of competing interest

The authors declare that they have no known competing financial interests or personal relationships that could have appeared to influence the work reported in this paper.

## Data Availability

Data will be made available on request.

## Appendix A Accreditation Components and Indicators

Learning Outcome

1. Students demonstrate discipline behavior.
2. Students demonstrate religious behavior at school.
3. Students demonstrate resilience and responsibility.
4. Students are free from bullying 
5. Students demonstrate 21st Century communication skills.
6. Students demonstrate 21st Century collaboration skills.
7. Students demonstrate 21st Century critical thinking and solving problem skills.
8. Students demonstrate 21st Century creativity and innovation skills.
9. Students demonstrate the ability to express themselves and be creative in enhancing their interest and talent.
10. Students demonstrate the ability to improve their learning performance.
11. Stakeholders are satisfied with the quality of the graduates.l
12. Graduates of recent year have certificate of competence
13. Graduates have secured employment or started their entrepreneurship.

Learning Process

1. The learning process enables students to develop their higher-order thinking skills actively and effectively.
2. Assessment of learning process and outcome is used as a basis for improvement and is implemented systematically.
3. Remedial and/or enrichment programs are provided to students who need them.
4. Students actively participate in learning and the classroom learning atmosphere is enjoyable.
5. Teachers enhance reading and writing literacy.
6. Teachers create a learning atmosphere that pays attention to safety, comfort, cleanliness, and makes it easy for students to learn.
7. The facilities and infrastructure available at the school are utilized optimally in the learning process.
8. The school organizes a production unit/business center/technopark.
9. Students carry out field work practices.

Teacher Quality

1. Teachers develop active, creative, and innovative learning plans by optimizing the environment and utilizing ICT or other methods appropriate to the context.
2. Teachers conduct self-evaluation, reflection, and competent development to improve performance periodically.
3. Teachers conduct continuous professional development to improve knowledge, skills, and insight
4. Teachers develop creative and innovative learning strategies, models, methods, techniques, and media.
5. Teachers carry out competency assessor training activities or internships in the world of work.

School Management and Governance

1. The school develops, socializes, implements, and evaluates the school's vision, mission, and goals.
2. The principal demonstrates academic supervision competency to help teachers understand and apply quality learning.
3. The principal consistently, participatively, collaboratively, transformatively, and effectively leads teachers, education personnel, and students to develop creative and innovative ideas to achieve shared vision, mission, and goals.
4. The school builds communication and interaction between school residents, parents, and the community to create internal and external harmony of the school
5. The school practices a safe, orderly, clean, and comfortable learning environment.
6. The school involves parents and the community from various backgrounds in the planning, implementation, and supervision of programs and activities.
7. The school develops, implements, and evaluates the implementation of the curriculum systematically, creatively, innovatively, and effectively.
8. The school implements effective, efficient, and accountable management of teachers and education personnel
9. The school implements good management of resources and infrastructure to support a quality learning process.
10. The school manages the income and expenditure budget transparently and accountably.
11. The school organizes student activity development to develop students' interests and talents.
12. The school provides student guidance and counseling services in the personal, social, academic, further education, and career fields to support their achievements.
13. The school carries out Internal Quality Assurance every year
14. The school has collaboration with the world of work
15. The school uses the practicum facilities effectively and efficiently
16. The school uses the vocational learning facilities effectively and efficiently.
17. The school manages a Career Center which aims to provide job vacancies, information services, recruitment, distribution, and placement of graduates.
